# Beetle and plant arrow poisons of the Ju|’hoan and Hai||om San peoples of Namibia (Insecta, Coleoptera, Chrysomelidae; Plantae, Anacardiaceae, Apocynaceae, Burseraceae)

**DOI:** 10.3897/zookeys.558.5957

**Published:** 2016-02-01

**Authors:** Caroline S. Chaboo, Megan Biesele, Robert K. Hitchcock, Andrea Weeks

**Affiliations:** 1 Division of Entomology, Biodiversity Institute and Department of Ecology and Evolutionary Biology, 1501 Crestline Drive, Suite 140, University of Kansas, Lawrence, KS, 66045, U.S.A.; 2 Kalahari Peoples Fund, 4811-B Shoalwood, Austin, TX, 78756, U.S.A.; 3 Department of Anthropology, University of New Mexico, Albuquerque, NM, 87131, U.S.A.; 4 Ted R. Bradley Herbarium and Department of Biology, George Mason University, Fairfax, VA, 22030, U.S.A.

**Keywords:** Hunting, indigenous knowledge, ethno-entomology, Bushmen, arrows

## Abstract

The use of archery to hunt appears relatively late in human history. It is poorly understood but the application of poisons to arrows to increase lethality must have occurred shortly after developing bow hunting methods; these early multi-stage transitions represent cognitive shifts in human evolution. This paper is a synthesis of widely-scattered literature in anthropology, entomology, and chemistry, dealing with San (“Bushmen”) arrow poisons. The term San (or Khoisan) covers many indigenous groups using so-called ‘click languages’ in southern Africa. Beetles are used for arrow poison by at least eight San groups and one non-San group. Fieldwork and interviews with Ju|’hoan and Hai||om hunters in Namibia revealed major differences in the nature and preparation of arrow poisons, bow and arrow construction, and poison antidote. Ju|’hoan hunters use leaf-beetle larvae of *Diamphidia* Gerstaecker and *Polyclada* Chevrolat (Chrysomelidae: Galerucinae: Alticini) collected from soil around the host plants *Commiphora africana* (A. Rich.) Engl. and *Commiphora angolensis* Engl. (Burseracaeae). In the Nyae Nyae area of Namibia, Ju|’hoan hunters use larvae of *Diamphidia nigroornata* Ståhl. Larvae and adults live above-ground on the plants and eat leaves, but the San collect the underground cocoons to extract the mature larvae. Larval hemolymph is mixed with saliva and applied to arrows. Hai||om hunters boil the milky plant sap of *Adenium bohemianum* Schinz (Apocynaceae) to reduce it to a thick paste that is applied to their arrows. The socio-cultural, historical, and ecological contexts of the various San groups may determine differences in the sources and preparation of poisons, bow and arrow technology, hunting behaviors, poison potency, and perhaps antidotes.

## Introduction

Archery appears relatively late in human history and is thought to represent a cognitive shift in human behavior, social organization, and tool-making in the Middle and Late Stone Age (Sisk and Shea 2009; Lombard and Phillipson 2010; Wadley 2011, 2013). Hunting requires great knowledge in observation, intelligence, planning, and skills; hunting technologies also require intensive attention to the environment, materials, learning, and skills (Lombard 2015). Though inadequately studied and poorly understood, the use of poisons on arrows to increase lethality must have occurred shortly after early humans developed bow hunting methods. Today, archery persists in a few indigenous communities around the world, e.g., the Pumè in Venezuela (Greaves 1997); Haddad in Chad (Nicolaisen 2010); Ache in Paraguay (Hill and Hurtado 1989), Hadza in Tanzania (Woodburn 1970; Marlowe 2010), some native North American Indians; and various San communities in southern Africa. Although archery was likely very important to the biological and cultural evolution of humans (Shea 2006; Backwell et al. 2008; Evans 2012) we are losing the opportunity to understand this crucial period because these ‘hunter-gatherer’ groups, with men responsible for hunting animals and women responsible for gathering fruits, nuts, berries, and tubers, have become progressively more sedentary and the ancient practices are disappearing worldwide.

**Figure 1.**
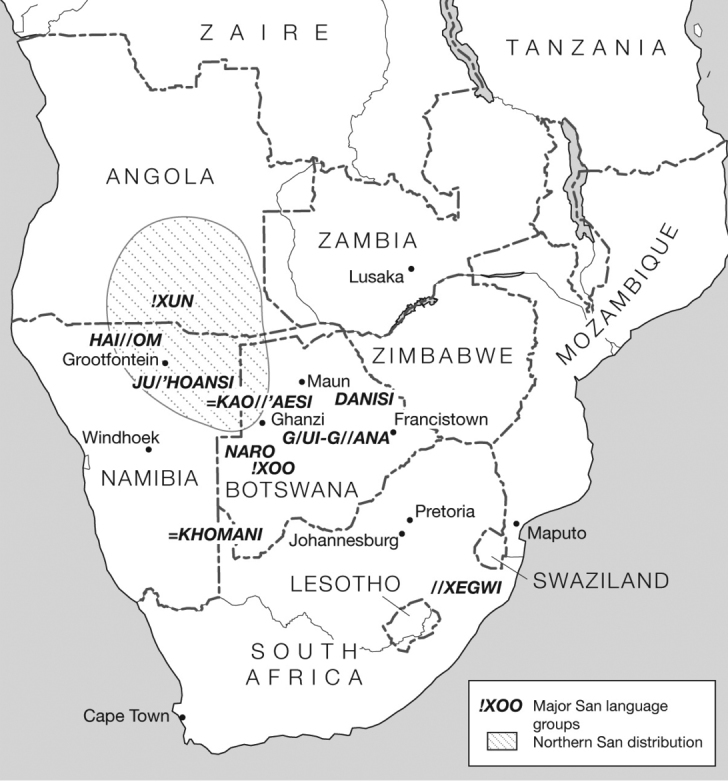
Map showing contemporary distribution of major San groups in southern Africa (prepared by Marieka Brouwer Burg).

This paper concerns the arrow poisons used by the Southern African San. The term San (also known as Khoisan, Basarwa, or “Bushmen”) covers many indigenous groups using so-called ‘click languages’ in southern Africa (Barnard 1992; Smith et al. 2000; Hitchcock et al. 2006; Fig. 1; see our Materials and methods below for further notes on San nations). In Africa, the origin of projectile points currently is dated to ~64,000–60,000 years ago, with the oldest from Sibudu Cave, South Africa (Lombard and Phillipson 2010; Lombard 2011). Some of the earliest reported arrowheads are carved bone projectile points that date to ~24,000 years ago, excavated from Border Cave, South Africa (d’Errico et al. 2012; Mitchell 2012). Ancient African rock paintings provide evidence of the use of arrows for hunting and conflict (Vinnicombe 1976). Poison arrows are discussed in ancient mythologies (e.g., Odysseus’ hellebore-poisoned darts in The Iliad, Homer ~800 BCE, see Scott 1923 and Cupid’s arrow in Ovid’s Metamorphoses, see Kline 2000) and in ancient religious texts (e.g., the Rig Veda from India (1100–1700 BCE), see Bisset and Mazars 1984). Theophrastus documented use of poison arrows in Africa in the 4^th^ century (Sharples, Huby, and Fortenbaugh 1995).

The most commonly used poisons across cultures are extracts from single plants or mixtures of plants (Neuwinger 1996, 1998). South American Indians in the Orinoco Basin use curare, extracted from plants (Loganiaceae: *Strychnos* L.; Menispermaceae: *Chondrodendron* Ruiz et Pavón, *Sciadotenia* Miers (Wintersteiner and Dutcher 1943). Poison dart frogs (Anura: Dendrobatidae) are used by the indigenous Chocó in Colombia (Myers et al. 1978); the frogs secure poison from their insect, mite and millipede prey (Clark et al. 2005; Daly et al. 2005).

Wikar (1779) is the first report of arrow poisons in southern Africa, one made from a “poison worm” by Hottentots along “the Great River” (called Groote River then and the Orange or Gariep River today), and the other made from a milky sap extracted from a tree (*Euphorbia virosa* Willd, Euphorbiaceae). Hunting with poison arrows continues in southern Africa today among a number of different San groups (Fig. 1, Map). The San are a diverse set of peoples, estimated today at 113,000, residing in six southern African countries: Angola, Botswana, Namibia, South Africa, Zambia, and Zimbabwe (Hitchcock et al. 2006; Sapignoli and Hitchcock 2013; Lee 2013). Some San groups still practice limited hunting and gathering, depending on their living conditions, population densities, technology, and the legal context in which they are operating. San use several hunting techniques: pursuit hunting on foot with bows and arrows, spears, or clubs; running down game animals and then dispatching them (“persistence hunting”; see Attenborough 2002; Liebenberg 2006, 2007); hunting from ambush, sometimes with bows and arrows or spears; mounted hunting, usually on horses but sometimes on donkeys, driving animals toward waiting hunters; and hunting with dogs. Noli (1993) provides a comprehensive review of archery in southern Africa. Hunting with guns is rare among most San. In general, the San hunt small game with traps and snares and use poison arrows on large game—antelope, buffalo, cheetah, eland, elephant, gazelle, giraffe, impala, lion, puku, springbok, warthog, wildebeest, and zebra (Campbell (1964, 1968a, b; Parker and Amin 1983; Marks 1977).

Schapera (1925) reviewed the plants, snakes, spiders, and two beetles, *Diamphidia simplex* Péringuey (now *Diamphidia nigroornata* Stål) and *Blepharida evanida* (Baly) (now *Blepharida vittata* (Baly), used for Bushmen arrow poisons; he noted geographic variation in poison formulae and that only the Kalahari Bushmen used the beetles. John Marshall (1958a: 378) reported, “There are four kinds of poison—a root which is rarely used, two grubs, and the pod of a tree. One of the two kinds of grubs is the larva of an unidentified beetle that lives in a bush; the other is the larva of *Diamphidia simplex* (now *Diamphidia nigroornata*) that lives in certain marula trees (Anacardiaceae: *Sclerocarya birrea* (A. Rich.) Hochst.). The beetle’s identity, however, is complicated by the presence of still a third beetle that apparently lives on the grub of *Diamphidia* complex…Lastly, still a fourth insect—small and hairy….” This third beetle has been identified as *Lebistina* Motschulsky (Carabidae: Lebiini) whose larva is a parasitoid, killing off the host chrysomelid larva as it develops. Koch (1958) indicated the involvement of multiple insect species belonging to various genera and families; he wrote that “results of all previous knowledge.....will have to be revised” (p 53). During the last 200 years, various sources of poison have been implicated (Table 1). Ethnographic data from Ju|’hoansi residing at Tsodilo in the period 1960–2013 indicate that Ju|’hoan hunters used *Diamphidia* poison mixed with *Sansevieria* juice (Robbins et al. 2012:9, Fig. 2). Robbins et al. (2012:15) summarizes ethnographic research that arrow poisons are derived from the pupae of three beetles that feed on *Commiphora africana* (A. Rich.) Engl. (Burseraceae) plants—*Diamphidia nigroornata* (Stål), *Diamphidia vittalipensis* [sic] (correct name *Diamphidia vittatipenis* Baly), and *Diamphidia formalis* [sic] (correct name *Diamphidia femoralis* (Gerstaecker)).

**Figures 2–7.**
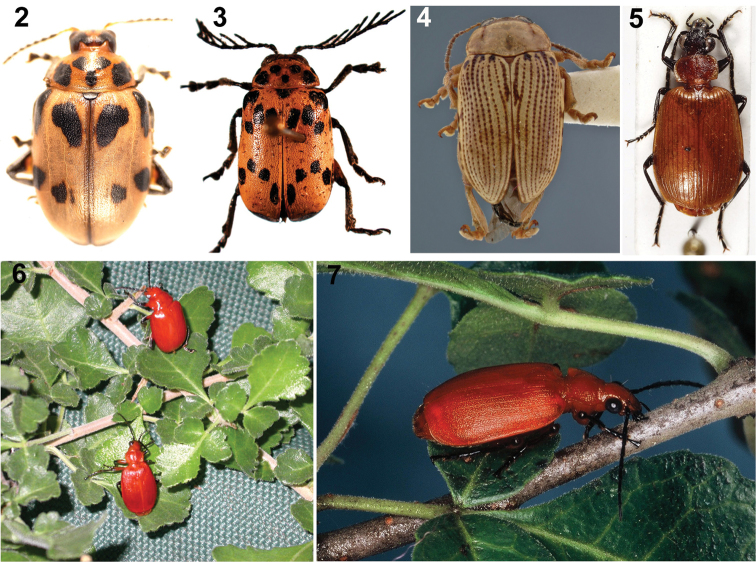
Arrow-poison beetles of the San people and their host plants (photos: CS Chaboo, or indicated if otherwise). **2** *Diamphidia nigroornata* Ståhl (=*Diamphidia simplex* Péringuey, =*Diamphidia locusta* Fairmaire), Namibia (Chrysomelidae) **3** *Polyclada* sp. (Chrysomelidae) **4** *Blepharida* sp., Kenya (photo: C Smith, USNM) **5** *Lebistina* sp. (Carabidae) **6** *Diamphidia femoralis* (above) and its predator-parasitoid enemy, *Lebistina* (below), on *Commiphora* plant in South Africa (photo: K Ober) **7** *Lebistina sanguinea* (Boheman) adult beetle on a *Commiphora* plant in South Africa (photo: E. Grobbelaar, SANC, ARC-PPRI).

**Table 1.** Summary of southern African San groups using poisons on hunting arrows and the source of the poison.

Indigenous group	Location	Poison (by genus)	Source/Researcher
Basarwa (Naro, G|ui, G||ana, !Ko, !Xóõ)	Bostwana: Ghanzi; Namibia	Beetle: *Diamphidia nigroornata*	De la Harpe et al. 1983; Woollard et al. 1984; Woollard 1986; Kann 1989
	Botswana	Beetle: *Diamphidia nigroornata*	Koch 1958; Heinz 1966, 1973; Steyn 1971; Heinz and Maguire 1974; Main 1987; Woollard et al. 1984; Woollard 1986
Batwa	Central African Congo Basin	Plants: *Eythrophylaeum guineense* G.Don (Caesalpiniaceae), *Palisota barteri* Hook (Commelinaceae) *Combretum* sp. (Combretaceae)	Parke 1888; Holmes 1888
“Bushmen”	Karoo	Plant: “black wax” [description appears to match *Adenium* poison]	Stow 1905
	Namibia: Grootfontein	Beetle: *Diamphidia simplex* (now *Diamphidia nigroornata*)	Händel and Gildemeister 1912
	Namibia	Beetle: *Lebistina* sp.	Koch 1958; Mebs et al. 1982
G|ui (=Gcwi)	Botswana: Central Kalahari Game Reserve	Beetle: *Diamphidia simplex* (now *Diamphidia nigroornata*)	Campbell 1968a; Silberbauer 1972, 1981a; Nonaka 1996
	Botswana: Central Kalahari Game Reserve	Beetle: *Polyclada flexuosa*	Marshall Thomas 1959; Gakelebone, pers. comm.; Sesana pers. comm.
G||ana	Botswana: Central Kalahari Game Reserve	Beetle: *Diamphidia simplex* (now *Diamphidia nigroornata*)	Campbell 1968a, b; Nonaka 1996; Gakelebone, pers. comm.; Sesana pers. comm.
G||olo	Bostwana: Central Kalahari Game Reserve	Beetle: *Diamphidia simplex* (now *Diamphidia nigroornata*)	Silberbauer 1981a, pers. comm.
Hai||om (=Heikum)	Namibia: Etosha Nat. Pk.	Plant: *Adenium bohemianum* (tuber)	Dieckmann 2007; this paper
Hottentots	? Kaukauveld	*Beetle: Diamphidia* spp.	Trommsdorff 1911
Ju|’hoansi (= !Kung)	Namibia: Otjozondjupa	Beetle: *Diamphidia* sp.	Böhm 1897; Marshall 1958a, 1976; Lee 1979; Mebs et al. 1982; de la Harpe et al.1983; Green 1998; Chaboo 2011; Robbins et al. 2012
	Namibia: Nyae Nyae	Beetle: *Polyclada flexuosa*	Oswalt 1973; Green 1998; Leffers 2003
	Namibia: Nyae Nyae	Beetle: *Diamphidia simplex* (now *Diamphidia nigroornata*)	Schapera 1925; Breyer-Brandwijk 1937
	Namibia: Nyae Nyae	Beetle: *Blepharida evanida*	Lewin 1912a, 1923
	Namibia: Gobabis	Beetle: *Diamphidia nigroornata*, *Diamphidia simplex*	Steyn 1957; Bijlsma and De Waard 1957
	Botswana	Beetle: *Diamphidia* sp.	Robbins et al. 2012
	Botswana	Beetle: *Diamphidia* sp.	Starcke 1897
	Botswana: Tsodilo	Beetle: *Diamphidia nigroornata*, *Diamphidia vittalipensis* [sic] (=*Diamphidia vittatipennis*), *Diamphidia formalis* [sic] (=*Diamphidia femoralis*)	Robbins et al. 2011
Kua	Bostwana	Beetle: *Diamphidia nigroornata*, *Diamphidia* spp.	Vierich 1981; Hitchcock 1982
		Beetle: *Diamphidia* spp.	Bartram 1997; Hitchcock & Ebert pers. obs.
		Beetle: *Polyclada* sp.	Valiente-Noailles 1993
		Beetle: *Diamphidia simplex* (now *Diamphidia nigroornata*), *Diamphidia nigroornata*	Valiente-Noailles 1993; Bartram 1997
Naro (=Nharo, Naron)	Botswana, Namibia	Beetle: *Diamphidia* sp.	Bleek 1928; Steyn 1971; Guenther 1986; Alan Barnard, Mathias Guenther, Maria Sapignoli, pers. comm.
Shua	Botswana, Zimbabwe	?	This paper
Tshwa	Botswana, Zimbabwe	?	This paper
Tsila	Botswana: Kweneng	Beetle: *Diamphidia nigroornata*	Vierich, pers. comm.
Valley Bisa	Zambia	Plant: *Acokanthera* sp.	Marks 1977
ǂX’ao-ǁ’aen (=Makaukau, Auen)	Botswana, Namibia	Beetle: *Diamphidia* sp.	Tobias 1957; Alan Barnard, Mathias Guenther, Maria Sapignoli, pers. comm.

Today, the San’s bow-and-arrow hunting and attendant tracking knowledge have a mythical status, but the facts of the poison sources and preparations are unclear. Several factors contribute misconceptions, outright errors, and ambiguous information about San arrow poisons. First, the use of the term “Bushmen” for diverse San tribes obscures apparent geographic variation in poison sources, recipes, and preparations. Second, insect taxonomists have rarely been involved in specimen identifications. Third, chemists analyzed specimens with presumed taxonomic identifications and left no specimen vouchers to confirm the species involved.

In this paper, we synthesize the anthropological, entomological, and chemical literature about San arrow poisons. Based on our fieldwork, we report arrow poison sources, their preparation and use, bow and arrow construction, and poison antidotes for the Ju|’hoan San in north east Namibia and the Hai||om San at Etosha National Park, Namibia. These two ethnic groups represent the largest San groupings in Namibia (Biesele and Hitchcock 2011; Dieckmann et al. 2014). We also summarize what is known about beetle poison use in seven other San groups—the G|ui, G||ana, G||olo, Kua, Naro, Tsila, and X’ao-ǁ’aen. We note arrow poison by one non-San group—Valley Bisa in Zambia (Marks 1977). Our paper supplements ethno-entomological documentation of the G|ui and G||ana in and around the Central Kalahari Game Reserve (see Fig. 1 map) and the Naro in Ghanzi, Botswana (Nonaka 1996).

## Materials and methods

**Figures 8–10.**
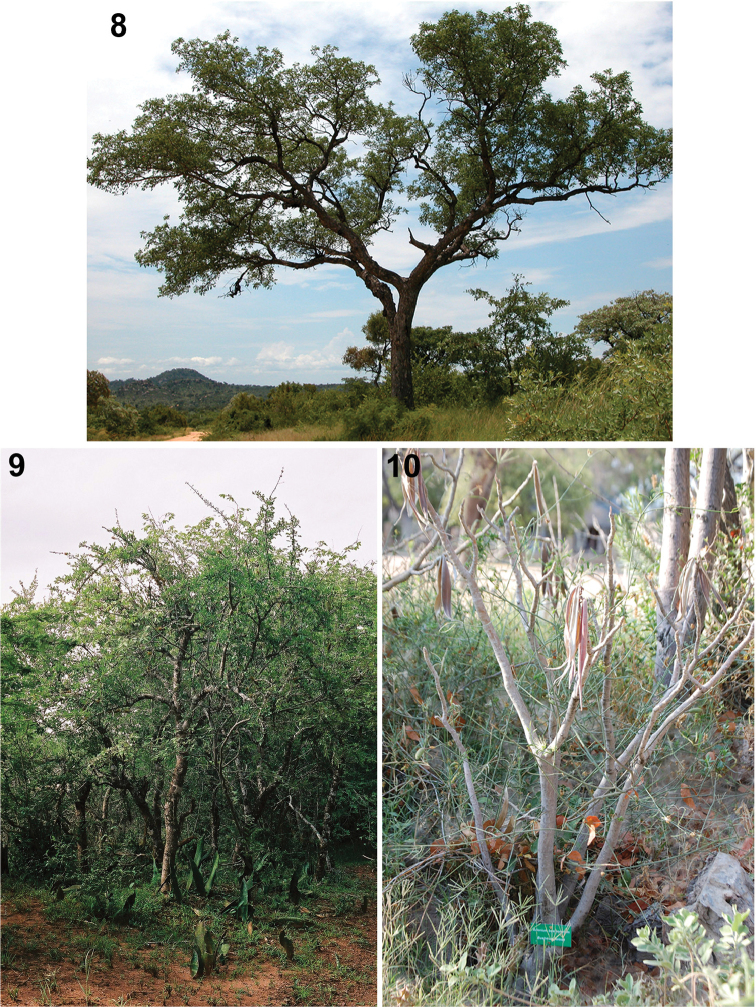
**8** *Sclerocarya birrea* (Anacardiaceae), South Africa (photos: CS Chaboo) **9** *Commiphora africana* (Burseraceae), South Africa, with *Sanseviera* (Dracaenaceae) at base (photo: E Grobbelaar) **10** *Adenium bohemianum* (Apocynaceae) used as arrow-poison by Hai||om around Etosha National Park, Namibia.

**Figures 11–17.**
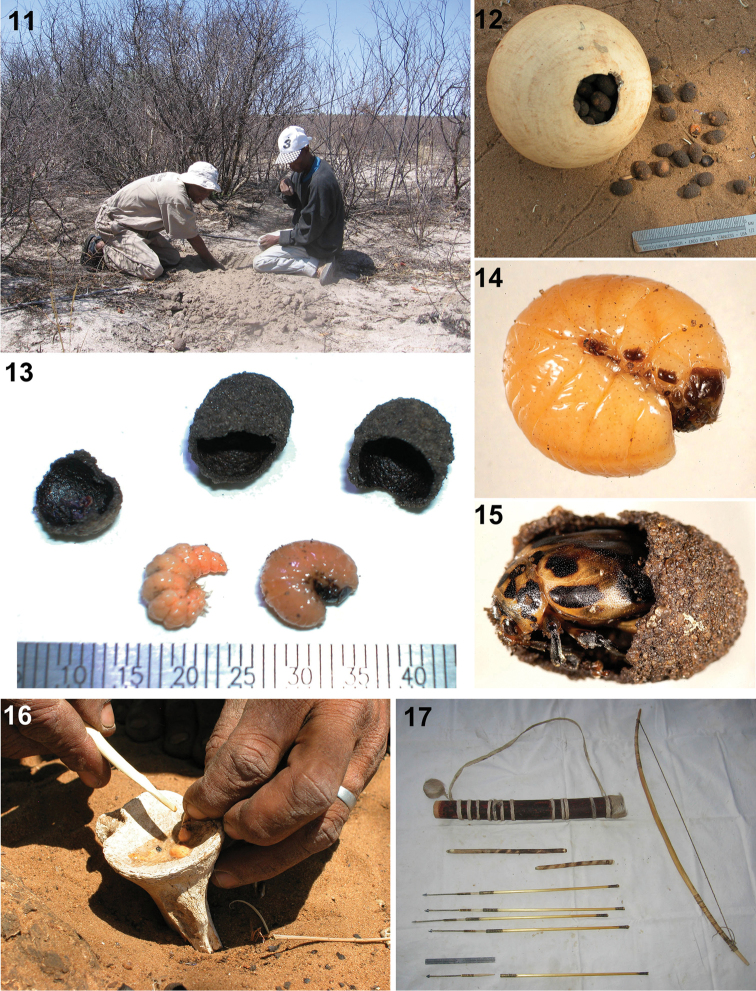
Arrow-poison beetles of the Ju|’hoansi, Tswumke Conservancy, Namibia (photos: CS Chaboo). **11** Typical collecting for beetle cocoons at base of a *Commiphora* shrub in the drip line **12** Ostrich egg-shell full of cocoons of Diamphidia nigroornata ab. locusta **13** Parasitoid carabid larva (left) and *Diamphidia* larva (right) extracted from collected cocoons **14** Cleaned 4^th^ instar *Diamphidia* larva extracted from cocoon **15** Adult *Diamphidia* beetle in cocoon **16** Squeezing the contents of leaf beetle larvae onto giraffe bone to prepare arrow poison **17** Typical hunting implements, quiver, bow, fire-sticks, and arrows.

We synthesize literature from anthropology, botany, chemistry, and entomology to develop a better picture of the arrow poisons used by the San in southern Africa. We present novel data and images (Figs 2–17) based on our collective field observations with two distinct San groups, the Ju|’hoansi in the Tsumkwe District of northeastern Namibia, and the Hai||om in northern and central Namibia (see Barnard 1992: 29–61 for Ju|’hoansi and Barnard 1992: 213–219 for Hai||om). Individual data is indicated by author initials [i.e., Caroline S. Chaboo (CSC), Andrea Weeks (AW), Megan Biesele (MB), and RK Hitchcock (RKH)]. Additional collecting of *Diamphidia* and *Polyclada* and their host plants, *Sclerocarya* (Fig. 8) and *Commiphora* (Fig. 9), was done by CSC and a colleague, E. Grobbelaar (Agricultural Research Council, Plant Protection Research Institute, Biosystematics Division), in South Africa.

*Symbols in San languages.* The San languages reported on here are characterized by sounds that are called clicks. This article is concerned primarily with the Ju|’hoansi and the Hai||om (see Lee 2013) and the UN-sponsored site, Written in the Sand, 2015). They have four clicks in their languages; the symbols for these clicks used throughout this text are as follows:

/ (|) Dental click as in G|ui

ǂ (‡) Alveolar click as in Nǂa Jaqna

! Alveopalatal click (as in !Kung)

// (||) Lateral click (as in G||ana).

San words for aspects of the poisons and their preparation are available in linguistic dictionaries (see Tanaka 1978; Dickens 1994; Visser 2003).

*Data collection.* In addition to the Ju|’hoansi, our literature survey revealed beetle poison use by seven other San groups—G|ui, G||ana, G||olo, Kua, Naro, Tsila, and ǂX’ao-||’aen. We summarize these data below. Insect inventories were conducted by CSC in expeditions to Botswana, Namibia, and South Africa (2005–2007), including a field study for three weeks with Hai||om and Ju|’hoan communities in Namibia in October 2007. Hitchcock worked among the Ju|’hoansi in 1987–2015 and Hai||om in 2011–2012. Biesele has extensive experience among the Ju|’hoansi from 1970 to the present.

*The landscape of our study.* Throughout this manuscript, we refer to the Kalahari. The Kalahari Desert is a basin-shaped plateau extending over northern South Africa, from eastern Namibia, across most of Botswana to southwest Zambia, and southern Angola (Encyclopaedia Britannica 2015). Our data is drawn from our experiences with different communities in different parts of this vast region and our map (Fig. 1) illustrates contemporary distributions of major San groups that have been studied in southern Africa. In this paper we focus mainly on the Ju|’hoansi and the Hai||om. The former are found in both Namibia (Tsumkwe District) and Botswana (North West District, Ngamiland), and the latter are found in Odangwa, Cunene, and Otjozondjupa Regions. Some groups are not shown because of the history of forced removals of people from their ancestral lands and the small population sizes. An accurate socio-linguistic map of historic and contemporary distributions of southern African peoples is fraught with problems but will be developed as our research expands.

The environmental settings of Nyae Nyae (Namibia) and Tsodilo-Dobe-/Xai/Xai (Botswana) is Kalahari sandveld and consists of tree-bush savanna and pans where water collects in the rainy season (Yellen and Lee 1976; Marshall 1976: 82–91; Lee 1979: 87–115; Thomas and Shaw 2010). The climate of the area is semi-arid, with a 4–6 month summer rainy season and moderate to cool winters with no rainfall (Yellen and Lee 1976; Thomas and Shaw 2010). Rainfall at Tsumkwe averages 450 mm per annum (range 219.8–627.8 mm; Namibia Meteorological Service data for 1984–2010). One of the unique features of this region is that there are between 15 and 25 permanent waterholes in the *molapos* (lowland areas) that lie between east-west trending sand dunes. Some of these waterholes are pans that are fed either by springs or by rainfall (Marshall 1976: 64; Yellen and Lee 1976: 36). As Yellen (1977: 21–22, 30–31) notes, the pattern in the distribution of dunes and *molapos* or *mekgacha* (low depressions between dunes) is important, as it provides different resource areas within relatively short distances that can be exploited by resident San populations.

*Interviews with Hai*||*om*, *Namibia.* Since 1954 the Hai||om have not been permitted to hunt in Etosha National Park, their original designated homeland. Subsequent displacement, settlement and shifting away from traditional ways have the consequence that hunters are rare and difficult to locate. Seven senior former hunters were interviewed within Etosha National Park, or on private farms south of Etosha, between 18–22 October 2007: Jan Tsumeb, Daniel Apia, Katison Khomob, Willem Dauxab, Fritz Khamuάb, Abram Geesep, and Jakes Kamaxάb. Interviews lasted several hours and involved a seated conversation and walks to collect specimens. Hunting nowadays is illegal; most informants said they were unaware of any active traditional hunters, but recalled their own hunting days or that of their fathers and uncles. Three elderly male Hai||om informants discussed their own hunting in their younger days, and pointed out traditional plants within the park. No poisons or arrows were prepared during the Hai||om interviews (this would have been illegal).

*Interviews with Ju|’hoansi, Namibia*. The Ju|’hoan communities together form a politically self-regulated body in the Nyae Nyae Conservancy. The region is part of the savanna biome, and is ecologically arid with brush or grass, almost no trees, and ranges from flat to gently hilly (Mendelsohn et al. 2009). Temperatures can be below freezing during winter and over 100°F in summer. The Ju|’hoansi live in small grass huts or rondavels (round adobe constructions with thatched roofs). Communities comprise 10–100 people living semi-permanently in some 36 villages around or near boreholes used for water. They are scattered throughout the area within one or more days of walking, the most common form of travel regionally. Today, men are still hunters where allowed and women are still the primary gatherers but more commonly both men and women carry out subsistence farming of maize, melons, beans, pumpkins, and other crops and some care for domestic animals and poultry.

The three-person field team, led by CSC, traveled to Tsumkwe (19°35’34.99 S, 20°30’07.99 E) in 2007, and then spent two weeks travelling to 10 villages scattered in the Nyae Nyae Conservancy. In each village, current and former hunters were interviewed, with questions presented by CSC in English and translated by team members (in German, Afrikaans, and Oshiwambo) or by native San speakers (three cases). Hunters are traditionally male; we did not encounter or hear of female hunters. Within 30 minutes of our arrival, most community members had surrounded our group, inserting comments from time to time, the senior women in particular correcting or debating details.

Twelve hunters were interviewed in Nyae Nyae Conservancy: Leon ‡oma Tsamkao, Tsumkwe Lodge; Trechie (‡Nlundi Village, Aha hills, 9–10 Oct 2007); Andreas (N|ama Village, 11 Oct 2007); Xushe Sao (N|ama Village, 11 Oct 2007); David Sao Iui (N|ama Village, 11 Oct 2007); /aice N!aucu (Xamsa Village, 11 Oct 2007); |aice ‡oma (Tambuti Village, 11.Oct.2007); G|aq’o ‡oma (Tambuti Village, 11.Oct.2007); N!aici Kaqece (Makuri Village, 12.Oct.2007); Kaqece Ikaece (Makuri Village, 12.Oct.2007); Il’ao N‡ao (Tsumkwe Lodge, 14.Oct.2007); and N!aici ‡oma (Tsumkwe Lodge, 14.Oct.2007). Interviews were conducted over one or two days. Each involved an initial interview, followed by a walk in the desert with the informants to look for the host plants and beetles; digging up beetle larvae (Fig. 11); returning to camp to prepare arrow poisons (Figs 16–17); and complete the interview. Each poison and arrow preparation session produced 10–20 arrows [>150 arrows observed in total]. Some reports indicate the Ju|’hoan word for the poison beetle is “Nga” or “N’gwa” (Livingstone 1858; Livingstone and Livingstone 1865; Schapera 1925; Breyer-Brandwijk 1937; Woollard et al. 1984), but our informants indicated that the word is “kua”.

Author MB has worked among the Ju|’hoansi since 1970, and served as director of the Nyae Nyae Development Foundation of Namibia from 1988–1992. She and RKH collected data about hunting and the use of poison from 46 people in 1987, 1992, 1995, 2001, and 2011–2014.

*Interviews with Ju|’hoansi, Bostwana.* RKH collected data from 56 interviews in Dobe, /Xai/Xai, Tsodilo in the years 1976, 1978–1982, 1985, 1988, 1992, 1995, 1997, 1999–2000, 2005, and 2011–2013. Hunters’ names are omitted here to protect their identities.

*Interviews with G|ui*, *G||ana*, *Kua*, and *Tsila*, *Bostwana*. RKH has conducted >140 interviews in about 15 visits (1976–2013) with these communities from the central and eastern Kalahari, many specifically on hunting. These data were collected in the Central Kalahari Game Reserve or in the resettlement sites outside the Reserve.

*Taxonomic identifications.* Due to the diversity of scientific names for plants and beetles used herein, our generic names are abbreviated to avoid confusion (Aca. = *Acacia*; Ade. = *Adenium*; Aco. = *Acocanthera*; Bob. = *Bobgunnia*; Comm. = *Commiphora*; Euph. = *Euphorbia*; Sans. = *Sansevieria*; Scl. = *Sclerocarya*).

*Hai||om poison plant.* Many former hunters indicated the poison plant on multiple separate occasions as *Adenium bohemianum* Schinz (Apocynaceae) (Fig. 10), which they call !*kores*. They confirmed the plant from photographs in our regional plant guides or by pointing out planted specimens in nearby gardens, including labeled plants in the educational garden of the Research Unit, Etosha National Park. Several members of the Apocynaceae are the source of latex, particularly from the roots, that are boiled and used as indigenous arrow poisons across Africa (Karimi 1973; Omino and Kokwaro 1993). For example, *Adenium obesum* (Forssk.) Roem. and Schult. is used as both a fish and arrow poison (Watt and Breyer-Brandwijk 1962; Gerhadt and Steiner 1986) and an undetermined *Adenium* species is used as an arrow poison by the Hadza of Tanzania (Bartram 1997). Plant taxonomic names used herein follow the online catalogue of plant names, PlantZAfrica (http://pza.sanbi.org/). The two chrysomelid host plants are identified as belonging to the Burseraceae, using Steyn’s (2003) key: *Commiphora africana* (A.Rich.) Engl. (poison-grub *Commiphora* which has true thorns) and *Commiphora angolensis* Engl. (sand *Commiphora* which lacks thorns).

*Beetle identifications and vouchers.* CSC obtained beetle specimens in several ways: receiving gifts of cocoons from informants from their stored supplies, collecting them with informants on bush walks, and purchasing containers from hunters’ stored supplies (e.g., Fig. 12). More than 5,000 cocoons were accumulated by CSC during fieldwork in Namibia; she also conducted nightly light-trapping to sample flying adults. Five hundred cocoons were dissected in the field to determine the beetle species and their life stages.

Beetle species were identified by examination of types and as part of a taxonomic review of specimens from museum collections in France, Germany, South Africa, and the USA. The chrysomelid beetle species in the Namibian Ju|’hoan arrow-poison case is *Diamphidia nigro*-*ornata* Ståhl (Figs 13–15; see Bryant 1942). Chrysomelid taxonomy follows Biondi and D’Alessandro (2012) who distinguished *Diamphidia* and *Polyclada* on the basis of antennal morphology—filiform in *Diamphidia* and pectinate or flabellate in males or serrate in females of *Polyclada*. Many synonyms for species of these genera appear in the historical literature. Heikertinger and Csikii (1940) is the last catalog of alticine flea beetles, but the last reviser of these species names was Bryant (1942) who indicates these synonyms for *Diamphidia nigroornata*: *Diamphidia lesnei* Achard, *Diamphidia locusta* Fairmaire, and *Diamphidia simplex* Péringuey.

Dissected cocoons revealed mostly larvae, some pupae, and some adults of leaf beetles. About 5% of the coccoons held only mature parasitoid carabid larvae (Carabidae: Lebiini; Fig. 13); but no adult carabids were found so we cannot be certain this is *Lebistina*. Carabid taxonomy follows the online catalog of Anichtchenko (2015).

Beetle vouchers are deposited in the collections of the National Museum of Namibia (NMWN, Windhoek), the American Museum of Natural History (AMNH, NYC), Agricultural Research Council, Plant Protection Research Institute, South African National Collection of Insects(ARC-PPRI, SANC, South Africa), and the University of Kansas Entomology collection (SEMC, KS, USA). John Irish, National Botanical Research Institute, Namibia, confirmed the identity of the plants.

## Results

Our cross-disciplinary synthesis of historial literature, reports of anthropologists, and our own collective fieldwork in southern Africa indicates that beetle arrow poison is used by seven San groups—the G|ui, G||ana, G||olo, Naro, Kua, and Tsila in Botswana, and the Hai||om in Namibia. Two San groups, the Shua and Tshwa from the north-eastern Kalahari of Botswana and Zimbabwe, do not use arrow poisons. One San group, the Hai||om, uses a plant poison. The Valley Bisa in Zambia is a non-San group that uses beetle arrow poison. Our research focused primarily on two large San groups, Hai||om and Ju|’hoansi, in Namibia but we assemble data for other smaller communities. Kiema (2010: 22–36) used the generic term Kua to refer to G|ui, G||ana, G||olo, Tsila, and all of the groups in the Central Kalahari region (our Fig. 1 map does not indicate a location of the G||olo as we have not found maps with this information). We follow the usage of Hitchcock (1978), Vierich (1981), and Valiente-Noailles (1993) who restrict the term Kua to the people of the eastern and southeast parts of the Central Kalahari. Since these diverse San nations are so poorly known, we briefly summarize the location, contemporary status, and knowledge of arrow poisons below.

*The G|ui and G||ana*, *Botswana* (Fig. 1). They live nowadays in the Central Kalahari Game Reserve, Botswana. Both groups have been the subject of forced evictions after the Botswana government removed them from the Central Kalahari in 1997 and in 2002 (Hitchcock et al. 2011). Modern G|ui and G||ana, have become progressively more sedentary (Osaki 1984, 2001; Ikeya 2001; Tanaka 2014). Some do still hunt with the aid of bows and poisoned arrows, although this has decreased significantly due to governmental restrictions. Nonaka (1996) report that G|ui, G||ana, and Naro use *Diamphidia simplex* (= *Diamphidia nigroornata*) for their arrow poison, but it is not clear how she identified the beetle species.

Campbell (1968a) briefly described G|ui and G||ana hunting with poison arrows. The arrows were lightweight, constructed in three detachable sections, and with little flight that necessitated the hunter getting close to his target. Campbell (1968a) indicated that poison was obtained from the pupae of *Diamphidia simplex* (= *Diamphidia nigroornata*), which were crushed and glued to the sinew around the haft of the arrow with plant gum. The arrow head was then dried over hot coals. This may be the earliest record of beetle poison use by G|ui. Campbell (1968a, b) reported that fresh poison worked faster, but that arrows were only active for about six months. Since the beetles are supposedly available seasonally (~2 months), the G|ui apparently had no poison for part of the year. Silberbauer (1965, 1972, 1981a, b) provided more details about the G|ui—cocoon collection, poison from the larvae of *Diamphidia simplex* (= *Diamphidia nigroornata*), bow and arrow materials, and poison preparation. These broadly resemble those used by other San, but certain details raise doubts. We note the extended life cycle of *Diamphidia*, with a long underground phase, which permits digging up larvae and pupae at any time of the year; this cast doubt on Campbell’s reports (1968a, 1968b) that G|ui had no poison for part of the year. We also wonder about ‘flaming the arrowhead’, which should theoretically deactivate certain toxins (Nonaka 1996).

Thomas (1959: 94–97) described the arrow poison preparation of people she terms the Gwikwe (=G|ui). The poison was derived from grubs, extracted from underground cocoons; she drew attention to the similarity of the life cycle with that of *Diamphidia simplex* (= *Diamphidia nigroornata*), as used by the Ju|’hoansi. She described two different colored pupae in these cocoons, which the Gwikwe regarded as male (a small, yellow, black-headed pupa with poison only in the legs) and female (a larger orange pupa with poison throughout the body). She may have been observing different stages of maturity of the 4^th^ instar larva of the chrysomelid and the *Lebistina* parasitoid. This poison was applied directly to the arrow shaft.

In the Central Kalahari, according to our G|ui and G||ana informants, the beetle that is used for arrow poison is *Polyclada flexuosa* (Baly). The larvae and adults feed on the leaves of marula trees (*Sclerocarya birrea*) (Jumanda Gakelebone, Roy Sesana, pers. comms. 2011–2013); pupation is likely to be in the soil around the host as in other species we have studied.

*The Kua*, *Botswana* (Fig. 1). The Kua (~7,500 people) practice part-time foraging and sedentary agro pastoralism; many work as herders with local cattle owners, and are dependent on government drought feeding and destitution relief programs (Vierich 1981). Their hunt involves poison from the “nymph” of *Polyclada* associated with *Sclerocarya birrea* (Valiente-Noailles 1993) and of *Diamphidia nigroornata* known from *Commiphora africana* (Valiente-Noailles 1993; Bartram 1997). Valiente-Noailles (1993) described Kua poison preparation, with 8–12 grubs squashed together with saliva on a mortar, then smeared unto the binding behind the arrowhead. The arrow was then air-dried. Bartram (1997: 326–327) wrote only of a *Diamphidia* species from a *Commiphora* host, but specified the rubbing of larval tissue directly onto the arrow shaft, which was then heated to dry. Observations of Kua in the eastern-Kalahari in 1975–1976, by Hitchcock and Ebert, revealed that they made poison from beetles and from ‘spider’s nests’ (Hitchcock and Ebert, field data); they assumed that the beetle identification was *Diamphidia nigroornata*. Vierich (pers. comm. 2014) indicates that alternative plants were used when beetles were unavailable, however she did not confirm the identity of these plants.

In the east-central Kalahari, arrow poisons were used until the late 20^th^ century. James Chapman, who visited Nkawane in this area in 1852, notes that the Bushmen there used bows, arrows and spears (Chapman 1971). He noted that the Bushmen killed elephants with spears, but he does not say whether or not poisons were used in elephant hunting. Based on observations made in 1850 in the eastern Kalahari, Schulz and Hammar (1897: 84) indicated that Bushmen used arrow poison “obtained from the juice of poisonous herbs and roots” and poisonous snakes.

*The Naro (Nharo, Naron), Botswana and Namibia* (Fig. 1). The Naro (~8,000 people) are a Khoe-speaking people that form the largest and most diverse set of San populations in western Botswana, stretching westwards into Namibia (Bleek 1928; Barnard 1976; Guenther 1986). They are located on the harder Ghanzi Ridge, but they are also found working on farms in northern Ghanzi and south of Ghanzi (Barnard 1992: 134–155). They use *Diamphidia* beetles for arrow poison (Bleek 1928; Steyn 1971; Guenther 1986; Nonaka 1996), but how the beetles were identified is not specified.

*The Tsila, Botswana*. The Tsila (~500 people) are found in the Central Kalahari Game Reserve, in the Northern Kweneng District and the eastern Central District. Vierich (pers. comm. 2014) observed their use of arrow poison derived from *Diamphidia nigroornata* and an unidentified plant.

*The Tshwa and Shua, Botswana and Zimbabwe*. Ethno-historic evidence suggests that poisoning of arrows is uncommon among these San groups, living in the northeastern Kalahari. Hodson (1912: 227) made the following observation “The Bushmen in this district are called Mashuakue, their headman being Kotama . . . They do not use the bow and poison arrow so common with Bushmen in the far Kalahari, but carry long assegaais [light spear], with which they stalk game. Some have rifles and are good shots at a close distance.” Detailed fieldwork with Tshwa and Shua (about 4,000 people), beginning in 1975, revealed that spears were the most common weapon used, along with clubs and guns of various kinds (Crowell and Hitchcock 1978; Hitchcock and Bleed 1997; Hitchcock et al. 2014). Tshwa who were forcibly moved to the east-central Kalahari in the 1950’s by Bamangwato cattle owners, picked up the use of bows and poisoned arrows from the neighboring Kua (Hitchcock 1978).

*The Valley Bisa, Zambia.* Marks reported on his 1973 observations of Valley Bisa hunts in Zambia (1977, pers. comm.). Hunters stalked up to large game before letting an arrow fly, a distance that was further than that for shotguns and even rifles. Their tactic was to stay slightly beyond the species ‘flight distance’ (distance before the prey flees), force the herd into a smaller space, and then arc the arrow to fall within the anticipated space. They did aim to get closer to some individual mammals (e.g., warthog). The arrows only had to lance the animal to get poison into the blood stream, but the wounded prey normally did not move very far away. Marks found that hunters used two different types of arrows for mammals and for birds, both poisoned with an extract made of pods and roots from an *Acokanthera* sp. (Apocynaceae; identified by biologists at University of Zambia; Marks 1977).

*The* ǂ*X’ao*-ǁ’*aen (=Makaukau, Auen), Botswana and Namibia.* The ǂX’ao-ǁ’aen San (~7,000 people) are sometimes called Makaukau or Auen (Schapera 1930: 33). They are found in the northern and southern parts of Ghanzi District (Groot Laagte, Kuke, D’Kar, Hanahai), Botswana and into the Omaheke region, eastern Namibia (Otjinene, Skoonheld, Donkerbos, Gobabis). The ǂX’ao-ǁ’aen use *Diamphidia* beetles for arrow poison (Maria Sapignoli, Job Morris, pers. comms., 2012, 2013, 2014).

*The Hai||om* (=ǂAkhoe) *in Namibia.* The Hai||om (11,000–15,000 people) is the largest and most widely distributed San population in Namibia (Widlok 1999; Dieckmann 2007; Koot 2013; Dieckmann et al. 2014; Hitchcock 2015). They Hai||om comprise different sociolinguistic groups according to linguists, anthropologists, and to the Hai||om themselves (Hahn et al. 1928; Dieckmann 2007: 112, Table 4.1) and generally speak the Hai||om language, part of the Khoe family of languages (Rapold and Widlok 2008). The Hai||om and ǂAkhoe were affected significantly by South West African and later Namibian policy relating to conservation and hunting. Etosha, which is considered by the Hai||om to be their ancestral homeland, was declared off limits in 1954 by the Department of Nature Conservation (Dieckmann 2007: 189–199; Suzman 2004; Friederich 2014: 60–69; Hitchcock 2015). Hunting laws restricted Hai||om and ǂAkhoe hunting practices. The vast majority of Hai||om today have mixed economic systems, combining a small amount of foraging with wage labor, some gardening, and food obtained from the government of Namibia as part of social safety net programs. Hunting is not done openly but relatively few Hai||om continue to hunt with bows and poisoned arrows (Widlok 1999; Dieckmann 2007; Peters et al. 2009; Kadison Khomob, pers. comm., 2012).

*The Ju|’hoansi in Namibia.* The Ju|’hoansi represent one of the earliest-diverging lineages of modern humans (Knight et al. 2003; Tishkoff et al. 2009; Mitchell 2010a; Pickrell et al. 2012; Schlebush et al. 2012) and are therefore some of the best-documented peoples on the planet as they have been studied intensively and over a long period, since the early 1950s (Marshall and Ritchie 1984; Marshall 2003; Marshall 1976: Lee and DeVore 1968; Lee 1968, 1978, 2013). Although they may not be the only, or the best model, of human’s hunting and gathering past, in some ways their history is very enlightening. Most Ju|’hoansi retain their language, culture, and many of their traditions till today. They have been able to secure some of their land and resources through advocacy, working with the Namibian government and non-governmental organizations (Biesele and Hitchcock 2011; Lee 2013). The Ju|’hoansi are the only indigenous people in Africa who still have the right to hunt for subsistence purposes using their traditional weapons. Some Ju|’hoansi continue to hunt with poisoned arrows, and they attempt to teach their young about finding poison materials, preparing the poison, and putting it on arrows. The Ju|’hoansi are, therefore, well-placed for the investigation of the use of arrow poisons.

*San hunting.* Bushmen tracking culture is well documented (Stander et al. 1997; Liebenberg 2000, 2001; Biesele and Barclay 2001). Indeed, San trackers were employed by opposing colonial militia in various conflicts (Lee and Hurlich 1982; Gordon and Douglas 2000; Guenther 2005). San participated also in fighting, sometimes using bows and arrows. One of the biggest fears of people with whom they had conflicts, such as settlers, was being struck by a poisoned arrow (Gordon and Douglas 2000; Hitchcock, Sapignoli, and Babchuk 2015). Lee (2003: 115) observed that a man hit with a poison arrow died, despite incisions made around the point of arrow entry to drain the poison. The most common weapon in family quarrels, suicides, homicides, and warfare has been poisoned arrows. The victim can die within one day if the wounded limb is not amputated (J. Marshall 1958b; newspaper citations). In addition to hunting for food, hunting has much prestige in the San community (Wiessner 2002; Marshall 1961, 1976; Lee 1972, 1979, 2013) and hunters are respected highly. Arrows are seen as having social and religious significance among 19^th^ and 20^th^ century San peoples in southern Africa (Wiessner 1983, 1984; Deacon 1992). Hunters learn how to make bows, arrows and poisons from older relatives (Wiessner 1983, 1984) and children use smaller bows and arrows in play. Today, poisoned arrows may be shared or traded among hunters (Lee 1984; L. Marshall 1976; CSC observations with Namibian Ju|’hoan informants) and are used as a ritual gift between husband and wife, who can form marital hunting partnerships (Biesele and Barclay 2001). Women can own arrows and thus, sometimes oversee meat distribution.

Hunts can last several days depending on the animal’s size and the slow paralysis by the poison. The tracks and spoor of the fleeing animal helps the hunter decide to immediately start stalking or return to the community to gather materials (e.g., water, food) and other men to help with the hunt. Animal tracks and dung inform the hunter about the size (size of footprint), species (nature of print), age (depth of foot print), wound (one side of foot prints heavier than other size), and travelling direction of the prey (Campbell 1968a; Liebenberg 2001; Lee 2003).

### Biology of arrow poison beetles

Beetles from the following genera appear in the literature as the source of a “Bushmen” arrow poison (Table 1):

Chrysomelidae (leaf beetles): Galerucinae: Alticini (flea beetles): *Blepharida*-group:

*Diamphidia* Gerstaecker (17 described species in genus; Biondi and d’Alessandro 2012; example Fig. 2)

*Polyclada* Chevrolat (16 described species in genus; Biondi and d’Alessandro 2012; example Fig. 3)

*Blepharida* Chevrolat (only from the subgenus *Blepharidina* Bechyné) (73 described species in genus; example Fig. 4)

Carabidae (ground beetles): Lebiini:

*Lebistina* Motschulsky (12 described species in genus; examples Figs 5–7).

Prathapan and Chaboo (2011) summarized the life cycle, biology and known host plants of the *Blepharida*-group. Blepharidines typically have large colorful adults (~1 cm); eggs are deposited in clusters on host plants, coated with fecal material; larvae that feed on host plant leaves, retain a fecal coat and eventually descend to the ground where they form a sandy underground cocoon; here they remain in a suspended larval stage, ready for pupation. The cycle from egg to adult can span 2–4 years; the underground phase is prolonged as a probable adaptation to unpredictable rainfall. Blepharidines tend to show generic-level specialization on certain plant families—Anacardiaceae, Apocynaceae, Burseraceae, Caesalpiniaceae, Clusiaceae, Eleaocarpaceae, Fabaceae, Lythraceae, Meliaceae, Moraceae, Theaceae, and Verbenaceae. Field reports of *Diamphidia* and *Polyclada* agree with the general blepharidine life cycle (Koch 1958; J. Marshall 1958a). Silberbauer indicated that the G|wi San were knowledgeable about some details of the beetle life cycle (larvae, migration to the drip line of host plants for pupation), but they were less informed about the adults, even mistaking them for shield bugs (Hemiptera: Pentatomidae; Silberbauer 1981). CSC confirmed that most cocoons are found at the drip line of *Commiphora* host plant shrubs in Namibia (Fig. 11).

*Arrow poison of the Ju|’hoansi, Nyae Nyae, Namibia. Locating host plants.* Informants indicated that they learnt about the locations of *Commiphora* host plants from older hunters. Once the low-branching *Commiphora* shrubs are located, the hunter initiates a new hole at the leaf-drop (=drip line) margin of the shrub; in some cases, ditches of previous digs (past years) were still apparent and our informant jumped into the 1m deep ditch and extended the ditch to encircle the plant. We observed some ditches forming a complete moat around plants. Ju|’hoan traditionally use a wooden digging stick (e.g., of the widespread Kalahari Christmas tree, *Dichrostachys cinerea* (L.) Wight & Arn. (Fabaceae) (Leffers 2003), but metal pipes are more commonly used today. Green (1998: 5) reported that Ju|’hoan hunters “take measures to protect Marula trees by building fire breaks around them during the dry season” and that the indigenous word for the larva is “!oan/aqro”. *Sclerocarya birrea* (marula) was indicated as the host plant of *Polyclada*, one of the arrow poison beetles (Leffers 2003), but CSC found none of these trees in the Nyae Nyae conservancy. *Collecting poison beetles*. The hunter sifts the loosened sand with his fingers, straining out the ~1 cm long, oval-shaped cocoons (Figs 11–13, 15). When sufficient cocoons have been collected into an ostrich egg shell (Fig. 12) or plastic container, the hunter returns home.

*Beetle poison preparation.* What follows is our typical observation compiled after 12 interviews with hunters who each made their poison with our observation. First, he arranges his tools, stabilizing an old giraffe or kudu knuckle bone with the concave surface facing upwards in the sand in front of him and placing the beetle cocoons nearby. A small fire is lit; traditionally a fire stick was used, usually made of *Commiphora pyracanthoides* Engl. (Burseraceae) (Leffers 2003), but nowdays a cigarette lighter is used. He breaks open a cocoon and taps out the single larva; non-larval forms (adults, pupae) are discarded. The larva is then rolled between his fingers, loosening the inner tissues from the integument. Using a stick as a pestle, he rubs hard against the skin to loosen tissue, then extracts it to mix on the bone mortar (Fig. 16); about 10 larvae are used per arrow. He chews the bark of *Acacia mellifera* (Vahl) Benth. (Fabaceae) to produce saliva which is mixed with the larval tissue and hemolymph. Published accounts of similarly chewed extracts list the plants used as: *Acacia mellifera*, *Asparagus* sp. (Asparagaceae), the bark of *Boscia albitrunca* (Burch.) Gilg & Gilg-Ben. (Capparaceae) (Leffers 2003), and *Ziziphus mucronata* Willd. (Rhamnaceae) (Roodt 1993). A bean of *Bobgunnia madagascariensis* (Desv.) J.H.Kirkbr. & Wiersema (Fabaceae) is heated over the fire, cooled, and added to the poison mixture. An unidentified toxic bean (J. Marshall 1958a) and the bean of *Bobgunnia madagascariensis* (Leffers 2003) appear as ingredients in some poison recipes. Ju|’hoan informants at |Xai|Xai in Botswana told Hitchcock that they use the juice of *Sansevieria* plants to improve the poison.

CSC observed that the ‘beetle paste’ of *Diamphidia nigroornata* larva is applied with a twig to the dried sinew that fastens the arrowhead to the wooden shaft; the hunter never touches the poison mixture. The arrows are then propped up against a log or hung up to air dry, and stored in a quiver made of the bark of the root of *Acacia luedertizii* Engl., False umbrella thorn (Fabaceae) (Leffers 2003). Finally, the hunter cleans his hands with loose sand. Different San groups squeeze *Diamphidia* beetle tissue directly onto the shaft of the arrow (see photographs in Campbell 1968a and Chaboo et al. 2007). Hunters indicated that cocoons or prepared poison arrows may be traded with hunters in other communities.

Literature sources reported that saliva made by chewing the bark of *Dicerocaryum eriocarpum* (Decne.) Abels (devil’s thorn) (Pedaliaceae) or the leaf of *Sansevieria aethiopica* Thunb. (Asparagaceae), are used to moisten the poison if it dries out (Leffers 2003: 87). The efficacy of the poison has been reported to last from three months (Green 1998) to two years (Clark 1975). Lee (1979: 137) reported that the high initial potency declines over time, and is essentially harmless after a year. Others report it to decline seasonally (Hitchcock et al. 2015).

*Ju|’hoan bow and arrows.* Arrows are constructed of grass reed (shaft), metal (arrowhead, blade), sinew for tying (from kudu), and glue (resin of *Acacia mellifera* obtained by damaging the bark) or beeswax (/aice ‡oma, pers. comm., Tambuti Village, 12 Oct. 2007). Bows are made from the wood of *Grewia flava* DC (Malvaceae). Leffers (2003: 34, 187) indicates that the shaft is from grass, *Andropogon gayanus* Kunth (Poaceae), and the glue is gum from *Terminalia sericea* Burch. ex DC. (Combretaceae).

*Preparation for the hunt.* CSC did not interview hunters about personal preparations before a hunt or special charms to accompany them. It is known that some rituals are performed to protect the hunter, improve his focus, and increase the hunt’s success. Leffers (2003) reports applications of plant extracts, including powders from roasted fruits and stems of Ceropegia distincta N.E.Br. subsp. lugardae (N.E.Br.) H.Huber (Apocynaceae), *Pavonia burchellii* (DC.) R.A.Dyer (Malvaceae) and chewing of plants (he also indicates a *Maerua* Forssk. sp. (Capparaceae)). He also reports avoidance of, or throwing sand at, plants of *Orbea huillensis* (Heirn) Bruyns (Apocynaceae), so as not to spoil the hunt (Leffers 2003: 150).

*Anti-venoms for beetle poison.* The following are considered as anti-venoms: a melon (informant Xushe Sao, 11 October 2008); liquid from *Sansevieria aethiopica* (Asparagaceae) (Leffers 2003: 170); and gemsbok cucumber, *Acanthosicyos naudinianus* (Sond.) C. Jeffrey (Cucurbitaceae) (Leffers 2003: 25).

*Beetle poison chemistry and effect.* After the earliest report by Wikar (1779) of insect and plant poisons and soon after by Paterson (1789) of snake and plant poisons, a century followed of murky identifications of the San poison. Almost 60 years later, Livingstone (1858: 189) added more details about the insect and plant poisons he observed; one involved a caterpillar (“N’gwa”) squeezed onto the arrow and allowed to dry and the other was milky sap of a *Euphorbia* L. (Euphorbiaceae) (cited also by Stow 1905). Andersson (1861) reported that poisoned arrows were used in fights with the Ovambo and Ovaquangari, in addition to hunting. Baines (1864) was the first to determine that the “caterpillar” reported by travellers was actually the larva of a beetle that the San squeezed unto arrows. Livingstone and Livingstone (1865) mentioned and illustrated arrows with plant and animal poisons of different peoples along the Zambesi. Passarge (1907) mentioned poison sticks, with “a lump of acacia gum drenched in arrow poison”; in this same text, he also reported two different poisons that were physically distinguishable as a dark-brown mass from a plant and light-brown dots of larval body fluid. Cornell (1920) mentioned Kalahari Bushmen arrow poisons derived from spiders, insects, a *Euphorbia* plant, and putrefying vertebrate corpses (death by lockjaw).

The next century saw different chemists examining residues on arrows or extracts of specimens sent to them and testing for hemolytic and toxic activity on various cells, tissues and live animals—fishes, frogs, birds (pigeons, sparrows), mice, cats, dogs, goats, rabbits, guinea pigs, and sheep (Böhm 1897; Starcke 1897; Heubner 1907; Händel and Gildemeister 1912; Lewin 1912a, 1923; Stigand 1913; Lewin 1923; Schapera 1925; Hall and Whitehead 1927; Pawlowsky 1927; Breyer-Brandwijk 1937; Steyn 1957; Bijlsma and De Waard 1957; Kündig 1978; De la Harpe and Dowdle 1980; Mebs et al. 1982; De la Harpe et al. 1983; Woollard et al. 1984; Kao et al. 1989; Jacobsen et al. 1990). Scientific attempts to verify the identity and nature of diamphotoxin, the identity of the beetles and their life stages, the host plants, and the recipes of poison preparation were unevenly documented, and led to further confusion about what was the poison being used by which San group. Schinz (1891) was first to observe the larval association with the host plant, *Commiphora africana*. He sent beetle specimens to the French coleopterist, Fairmaire, who described it as a new species, *Diamphidia locusta* (Fairmaire, 1893), which is now considered a chromatic form of *Diamphidia nigroornata* (see Bryant 1942). Later, Schultze (1907: 663) indicated “worms” at the roots of *Commiphora dinteri* Engl. Lewin (1912a) reported that Bushmen and Hottentots used a kind of caterpillar, combined with extracts of a *Euphorbia* species and Lewin (1912b) reported *Blepharida evanida* (Baly) and *Blepharidella lewini* Weise as possible sources of arrow poison. Rarely, a few field researchers provided more reliable information. For example, Trommsdorff’s (1911) fieldwork in “Kaukaufeld....with Hottentots” provided the first photographic illustration of three beetle species (adults and larvae) used for poison. No taxonomic identification was given in the text, but the adult beetles in their photograph plates have filiform antennae and are thus *Diamphidia* species. By 1864, the general view was that a beetle was involved (Baines 1864).

As chemists explored the nature of the poison, the taxonomy for the poison also evolved. Shaw et al. (1963) was the first major synthesis about the sources, preparation and the chemistry of ‘Bushmen’ arrow poisons. In addition to Steyn’s (1949) ~ 300 poisonous plants known from South Africa, they reported 16 different plant species in the genera *Acokanthera* G.Don, *Adenium* Roem. & Schult., *Euphorbia* L., *Haemanthus* L., *Hyaenanche* Lamb. & Vahl, *Pachypodium* Lindl., *Swartzia* Schreb., *Solanum* L., *Strophanthus* DC., and *Strychnos* L., and 15 different animal-derived arrow poisons, obtained from beetles, scorpions, spiders, and snakes that were used as arrow poisons by different ‘Bushmen’ groups from Central to southern Africa.

The beetle poison has been identified as a protein and referred to as a toxalbumin (Shinz and Böhm 1894; Böhm 1897; Starcke 1897; Breyer-Brandwijk 1937), diamphidia toxin (Breyer-Brandwijk 1937), and diamphotoxin (De la Harpe et al. 1983). Its effect has been explained as interfering with cell membrane by modulating calcium concentration (Kündig 1978), causing an influx of Ca^2+^ ions (Jacobsen et al. 1990); the affected animal exhibits massive hemolysis, convulsions, paralysis, then death (De la Harpe and Dowdle 1980; Mebs et al. 1982). Because of the high toxin concentration in the “pupa” compared to that in the adult, De la Harpe et al. (1983) suggested that diamphotoxin must have some functional role in this life stage, but it was unclear how autolysis was prevented. Diamphotoxin protein may be similar to toxic insect proteins found in tiger moths (Rothschild et al. 1970; Hsiao et al. 1980) and to leptinotarsin from the beetle, *Leptinotarsa* Chevrolat (Hsiao and Fraenkel 1969; Snyder 1971; Parker 1971, 1972; Satin et al. 1978; Hsiao 1978; McClure et al. 1980; Madeddu et al. 1985a, b; Miljanich et al. 1988). *Leptinotarsa* (subfamily Chrysomelinae) is phylogenetically distantly related to the galerucine *Blepharida*-group. The protein, leptinotarsin, also kills animals. It is unknown if leptinotarsin and diamphotoxin are related—evolutionarily, biogenetically, structurally, or in effects. Kann (1989) developed a protocol to purify diamphotoxin to facilitate the sequencing of its amino acid structure, but indicated the need to refine the protocol to obtain larger pure samples. At this point, a comprehensive and systematic approach to examining all life stages of all the *Blepharida*-group beetles for similar toxic activity will be useful to understanding the origin and biology of this remarkable protein.

*Is the Lebistina parasitoid a source of arrow poison*? This African carabid genus comprises 12 described species (Anichtchnko 2007–2014). Carabidae are commonly called ground beetles because they are generally ground-dwellers; however, *Lebistina* belongs to the tribe Lebiini, an evolutionary branch that has evolved a free-living first instar larva (technically called a triungulin). The lebiine first instar larva searches for host prey, attaches to it for feeding and in so doing becomes an ectoparasitoid which eventually kills its host (see Weber et al. 2008). Koch (1958) identified six different species of Coleoptera in the cocoons dug up by the “Bushmen” and suggested that the host plant, chrysomelid herbivores, and the *Lebistina* parasitoid formed trophic chains, where the “composition of the toxins and degree of toxicity may differ in each of these six [beetle] species”. It is remarkable, but not unknown, that some insects appropriate offensive or toxic chemicals from their prey. Koch’s (1958) concept has been perpetuated in the literature, implying that the San consider the parasitic *Lebistina* larva as more toxic than its chrysomelid host (e.g., Mebs et al. 1982; Lindroth 1971; Valiente-Noailles 1993; Neuwinger 1996). Koch (1958) indicated that only 1% of the chrysomelid cocoons collected contained a *Lebistina* larva. In our fieldwork, we dissected 500 *Diamphidia* cocoons and found mainly chrysomelids (many larvae, few pupae, few adults), few spiders, and only three carabid larvae (Fig. 13). Our Ju|’hoan informants used only chrysomelid larvae for poison preparation, discarding chrysomelid pupae and adults, and any other species. Chemistry assessments of *Lebistina* have not been done. Given the low level of parasitoidism that we observed, we conclude that it is very unlikely that the San use such rare larvae for poison and we will not discuss *Lebistina* as a poison source further.

### Arrow poison of the Hai||om, in and around Etosha National Park, Namibia

Andersson (1856) indicated that the Ovaherero used milky-white, gummy extracts of *Euphorbia candelabrum* as poison on their arrows, whereas the Hill-Damaras used these extracts to poison pools where game animals drank (e.g., buffalo, p. 242). Böhm (1890) analyzed an extract of *Adenium bohemianum* that was sent to him in Leipzig, Germany—identified as the arrow poison of the Bergdamara from north Damaraland and the Ovambo in Kaoko. The Bergdamara bought the plants from the Ovambo, who called it ‘exuja.’ Böhm (1890) extracted and crystallized a poisonous glucoside from this ‘exuja,’ for which he coined the term ‘echujin’ and described it as a genuine resin. He tested its effects on frogs, rabbits, cats, and a dog, all of which died. This led him to conclude that ‘echujin’ was a cardiac poison, like digitalis. Böhm’s (1890) report is the only one we found showing the trade or sale of poison between different ethnic communities of San.

Dornan (1925) and Schapera (1925) indicated that various “Bushmen” groups used several different plant species in the genera, *Acokanthera*, *Haemanthus*, *Buphane*, and *Euphorbia*, depending on geographic location. Livingstone and Livingstone (1865) mention poisoned arrows throughout their narrative and illustrated them (p. 466); they described the poison of natives in the upper cataracts of the Zambesi as an extract (“kombi) from the plant *Strophanthus* that felled most game except elephant and hippopotamus. We found Sands et al. (2011) to be the only recent indication of *Strophanthus* as a poison, used by the ǂ Hoan in the western Kweneng District, Botswana.

Hall and Whitehead (1927) studied the bows, arrows, and moving film of Hai||om, collected by CE Cadle on his 1925 Denver African Expedition. They reported the plant poison to be that from *Euphorbia*, but suggested that the Hai||om may use different poisons (p 55). They scraped the poison off the arrows and followed Fuller’s (1920) protocols for studying another deadly toxin, namely strophanthin. The extracts they prepared killed frogs and cats; further chemical analyses conducted targeted alkaloids and glucosides. They referred to the poison as “ouabain” but the reason for this is unclear as they did not conduct comparative chemistry with other ouabain arrow poisons, known to be widely used in Africa (Burton 1856; Castellani and Chalmers 1919; Clark et al. 1975; Maitai et al. 1973).

Our finding of *Adenium bohemianum* as the source of Hai||om arrow poison confirms three previous reports (Steyn 1957; Neuwinger 1996; Peters et al. 2009). Steyn (1957) indicated that the Bushmen he worked with were aware of the chrysomelid larvae, but considered this poison to kill prey too quickly. They apparently preferred the slower-acting poison from *Adenium bohemianum*, but Koch (1958) was unable to confirm this. Our Hai||om informants had never heard about the beetles and laughed about the “Bushmen” (Ju|’hoan) using such [silly] things. They also did not mention ouabain as a plant poison.

Given the findings of poison on hunting implements at Sibudu Cave, an intriguing data point comes from Stanford (1909) which may be the only account of poison prepration of the San living the Drakensberg Mountains, South Africa. The San chief prepared poison by boiling the root of a shrub with the bark of a tree in a clay pot for several days. The Drakensberg area has over 35,000 cave paintings (Mitchell and Smith 2009), but no San were believed to live there. Today, there is a group in the Drakensberg Mountain region, the Abatwa (Zulu word for San) who believe that they are descendants of the San who lived in the region in the 19^th^ century (Francis 2009; Prins 2009).

*Plant poison preparation.* Among the seven hunters interviewed, only one eventually admitted to hunting illegally and showed us his hunting gear—including poisoned arrows. According to our seven informants, tubers of *Adenium bohemianum* are dug out, cut into pieces, and the inner plant tissue is scraped into a cup using an animal bone. This is then boiled for “a long time” until it becomes a thick black glue that is applied to the arrows. In addition to our observations, other methods of poison preparation and application appear in the literature. Grubs are dried, ground and mixed with saliva or plant sap (Stigand 1913) or living larvae are squeezed to apply the entrails directly to the arrow-head (Breyer-Brandwijk 1937; Chaboo et al. 2007).

## Discussion

Our discussion is organized around three topics below: bow and arrow technology, Ju|’hoan beetle poison (source and pharmacology), and Hai||om plant poison (source and pharmacology).

*Comparison of Namibian San bows and arrows.* It is beyond the primary focus of this paper to discuss San arrow technology in detail, however it is important to pay attention to subtle aspects of design that might inform which poison source was adopted by the community. The use of poisons to increase the lethality of arrows and increase the success of a hunt must surely have impacted the design of bows and arrows, and therefore has implications for human cultural evolution. Hall and Whitehead (1927) found that the arrows of three San groups in Namibia—Hai||om, Ju|’hoan and Ovachimba (= ova-Himba) were made of different woods and other materials; they have different sizes and weights (Goodwin 1945; Clark 1975). The arrow poison was visible as a thin shellac on the Hai||om arrows, but not on the Ju|’hoan arrows. The Hai||om bows and arrows came in two sizes, both being larger and heavier than those of the Ju|’hoan. We found Ju|’hoan arrows to have a three-part construction, matching Hall and Whitehead’s (1927) description; our informants indicated that the metal arrowhead (as opposed to carved stone of past times) entered the prey, and the other two parts detached and fell to the ground on impact—thus notifying the hunter that his arrow had hit the prey. Marshall Thomas (1959) commented that the arrow release was different between the Ju|’hoansi and the Gwikwe, suggesting subtle differences in archery styles, however Deacon (1984) proposed that stylistic differences may be a modern phenomenon.

We observed some modern impacts on Ju|’hoan bows and arrows (Fig. 17). Wood and stone arrowheads were replaced a long time ago by arrowheads crafted from metal (Wiessner 1983; Robbins et al. 2012). We observed nails and fencing wires being pounded into arrowheads. In one community we saw an old can being used to mix the poison, instead of the traditional giraffe knuckle bone. Several hunters were using PVC pipe containers, instead of bark-derived quivers; Bartram (1997) also mentioned plastic quivers. Some hunters remarked on the efficiency of a gun, but indicated a preference for arrows because they were quiet and did not startle the animals, causing them to run away.

In their illustrated description of Hai||om arrows, Hall and Whitehead (1927) noted that there were two sizes made from the wood of *Grewia* (Malvaceae) or *Cordia* (Boraginaceae) (Peters et al. 2009), with partially stripped feathers on the shafts. Peters et al. (2009) reported three different-sized Hai||om arrowheads, one of which is poisoned with the boiled latex of *Adenium bohemianum*. None of the Hai||om we interviewed admitted to hunting with guns but Widlok (1999) reported instances of Hai||om poaching with guns on commercial farms along the northern border of the Etosha National Park. Gun hunting is not common among the Namibia San for various reasons—guns are hard to come by; hunting with guns is illegal in much of Namibia; it is difficult to obtain ammunition; and people often prefer quieter hunting methods.

*Source of beetle poison.* The life stages used by the San to obtain their poison have been reported as the larva and pupa, but all the past chemists who worked with material did not collect the specimens themselves and could not distinguish larva from pupa (e.g., De La Harpe et al.’s (1983) photograph of a “pupa” is actually a larva). Breyer-Brandwijk (1937) found that both larvae and cocoons show chemical activity, suggesting that the larva secretes poison in its cocoon. De la Harpe et al. (1983) also indicted lower concentrations of diamphotoxin in the adult. It is possible that all the beetle life stages have diamphotoxin, but the unevenness of historical studies raise uncertainty about both the identity of the beetle and the identity of the active ingredient. The synthesis of diamphotoxin is unexplored; chrysomelids could either sequester chemicals from their host plants, using them as precursors, or manufacture the chemicals *de novo*. Both Burseraceae and Anacardiaceae have diverse secondary chemicals (Daly et al. 2011; Pell et al. 2011) and have well-documented ecological interactions with blepharidine beetle species (e.g., Becerra 1993, 2004). The accuracy of past species determinations is questionable; none of the chemical work acknowledges how beetles were identified and no specimen vouchers were retained. Castellani and Chalmers (1919: 180) proposed the actual killing agent to be the microbes growing in the rotting larvae of *Diamphidia simplex* (= *Diamphidia nigroornata*), e.g., tetanus carried on arrows elsewhere (Hall and Whitehead 1927). These ideas, along with the confusing taxonomy of *Blepharida*, *Diamphidia*, and *Polyclada*, leave open questions about which species and genera are poisonous.

Based on our specimen collections of larval, pupal and adult stages (we did not collect egg stages) with the Ju’hoansi San at Nyae Nyae, we determine those beetles as *Diamphidia nigroornata*. Bryant (1942) indicates several species names as synonyms of *Diamphidia nigroornata*. Other researchers have photographed poison beetles used in the Nyae Nyae region and which are a different species from our sample. Thus, several *Diamphidia* species are used for poison.

Morphology-based revisions of these genera are now underway to test species concepts. Molecular methods are required to link different life stages with adults, to identify the larvae being used as poison. Linking the life stages and the host plants is crucial to clarifying which beetle is being used by which local San community.

*Effect and pharmacology of beetle poison.* The corpus of chemical studies of the last 200 years point to a highly toxic basic peptide, called a toxalbumin (Böhm 1897; Hall and Whitehead 1927), and diamphotoxin (coined by De la Harpe et al. 1983) that only works by entering the blood stream (Marshall 1958b: 379) and affects cell membrane permeability and electrolyte balance (e.g., De la Harpe et al. 1983), causes tissue hypoxia (Kao et al. 1989), neurotoxicity (Starcke 1897; Woollard et al. 1984), rapid and severe lysis of red blood cells, and hemoglobinuria (excessive loss of red blood cells through urine). One early outcome is a slow paralysis (Breyer-Brandwijk 1937) then death by renal failure, but the cause of death apparently varies according to the injection site, absorption rate, and dosage. Chemical analyses and equipment have changed a lot since many of the historical studies were done. Modern analyses and comprehensive targeting of the various beetles, along with the host plants, would help greatly in unambiguously answering outstanding questions and doubts about the San arrow poison beetles.

*Comparison of diamphotoxin and leptinotoxin.* The speed, impact, and lethal nature of diamphotoxin recalls another toxic leaf beetle molecule, leptinotoxin, isolated from adults of *Leptinotarsa haldemani* Rogers in North America (Chrysomelidae: Chrysomelinae) (Crosland 1982; Crosland et al. 1984; Maddedu et al. 1985a, b; Miljanich et al. 1988). Chemists working on diamphotoxin and leptinotoxin in the mid-1980’s were apparently unaware of each other’s work, and did not compare these two protein toxins from relatively closely-related beetle taxa. Leptinotoxin seems to target calcium channels and is neurotoxic. Diamphotoxin on the other hand kills by hemolysis with a combination of tissue hypoxia and neurotoxicity. A comprehensive modern chemical analysis must be done to discern any relationship in genesis, molecular structure, and mode of action between leptinotoxin and diamphotoxin.

Beetles are known for other potent chemistry. For example, cantharidin or “Spanish fly”, extracted from meloid beetles, *Lytta vesicatoria* (L.) (Meloidae) (inaccurately referred to as *Cantharis vesicatoria* (Cantharidae) in some publications), was known to the ancient Chinese and Greeks as an aphrodisiac (Karras et al. 1996; Moen et al. 2001). Such toxic chemicals in beetles play a central role in the formation of parasitic and mimicry relationships, e.g., between true *Cantharis* and *Lytta* and the secondary toxicity of their predators, such as poison frogs and birds (Dumbacher et al. 2004; Clark et al. 2005). Some predators appropriate offensive or toxic chemicals from their prey: dart frogs in Colombia (Myers et al. 1978), tiger keel back snake in Japan (Hutchinson et al. 2007), poison rat in Somalia (Kingdom et al. 2011), and poison birds in New Guinea (Dumbacher et al. 2004). As a parasitoid, *Lebistina*, could be sequestering chemicals of its hosts, *Diamphidia* and *Polyclada*; their similar body form and coloration certainly suggest a model of Müllerian or Batesian mimicry (Fig. 6).

*San ethno-entomology.* In addition to the beetle poison, the San collect and eat other insects, but reports are scattered. We did not conduct a complete ethno-entomological inventory of the Hai||om and Ju|’hoan as we believe that the degree of settlement and diversion from their traditional nomadic lifestyle would probably distort such data. However, we summarize here what other insects are used by the San. These are collected and eaten, dependent on seasonal outbreaks and swarming: certain caterpillars (e.g., Mopane worms (Saturniidae: *Gonimbrasia belina* Westwood = now *Imbrasia belina*) (Roodt 1993; Leffers 2003) and other lepidopteran species (Passarge 1907); grasshoppers (see Samuel Daniel’s 1805 painting in Preston (1989); Passarge 1907; Marshall J. 1958a; Marshall L. 1961; Osaki 2001); termites (Bleek 1928; Bjerre 1958; Peters et al. 2009); sugary lerps secreted by jumping plant lice (Psyllidae) (Livingstone 1858: 182); locusts (Peters et al. 2009); bee honey (Marshall Thomas 1959; Peters et al. 2009); click beetles (Lee 1972); and winged ants (Stanford 1909; Lee 1972; Marshall Thomas 1959). Some insects are eaten roasted—caterpillars and grasshoppers (Passarge 1907; Chaboo, pers. observ.) and termites (Bleek 1928). The African honeybee is aggressive, so honey collection is an activity that is approached with caution (Marshall Thomas 1959). Fieldwork is needed to determine the full selection of insects that different San groups utilize as food sources.

*Source of Hai||om plant poison.* The angiosperm genus *Adenium* Roem. and Schult. (Apocynaceae) comprises five species: *Adenium bohemianum* Schinz, *Adenium multiflorum* Klotzsch, *Adenium obesum* (Forssk.) Roem. and Schult., *Adenium oleifolium* Stapf, and *Adenium swazicum* Stapf. All species are limited to sub-Saharan Africa with the exception of *Adenium obesum* whose range extends into the Arabian Peninsula and Socotra. Four *Adenium* species, as currently circumscribed by Plazier (1980), have relatively narrow geographic ranges in southern or tropical East Africa (*Adenium bohemianum*, *Adenium multiflorum*, *Adenium oleifolium*, and *Adenium swazicum*). However, taxonomists prior to Plazier (1980) have treated three of these species as varieties of either *Adenium obesum* (*Adenium multiflorum*, *Adenium oleifolium*) or *Adenium bohemianum* (*Adenium swazicum*). Moreover, the wide distribution of *Adenium obesum* spurred a number of heterotypic species descriptions in the past and those names are now considered taxonomic synonyms (e.g., *Adenium honghel*, *Adenium somalense*) yet they remain in use, albeit incorrectly. Consequently, one may be prevented from verifying which *Adenium* species a particular ethnographer, natural historian or chemist reported or investigated, even if the identification was made correctly, due to the malleable taxonomic species concepts in this genus.

At least 29 different glycosylated cardenolides (“glycosides”) have been isolated from *Adenium* species (Yamauchi and Abe 1990), and some have well-documented effects on cardiac cells and are referred to as cardiac glycosides. These include, but are not limited to, digitalin (=gitoxigenin 3-*O*-glucosyldigitaloside), honghelin (=digitoxigenin b-d -thevetoside), and somalin (=digitoxigenin b-d-cymaroside). Like *Adenium* species names, cardenolide nomenclature contains synonyms that are used interchangeably by authors, making cross-comparisons a challenge (Harbourne et al. 1999). Phytochemical description of species may be overly general; for instance, stating that a species contains “digitoxigenin” does not indicate the component sugar that modifies the cardenolide and is not a precise chemical reference. Yamauchi and Abe (1990) reported two pregnanes from *Adenium obseum* (neridienone A and 16,17-dihydroneridienone); these steroid-derived compounds are similar to animal hormones such as cortisol.

The angiosperm genus *Acokanthera* G. Don (Apocynaceae) comprises five species: *Acokanthera schimperi* (DC.) Schweinf., *Acokanthera oppositifolia* (Lam.) Codd, *Acocanthera laevigata* Kupicha, *Acocanthera rotundata* (Codd) Kupicha, and *Acocanthera oblongifolia* (Hochst.) Codd. All species are limited to East Africa, with the exception of *Acokanthera schimperi*, which ranges from tropical East Africa into the Arabian Peninsula. *Acokanthera oppositifolia* ranges from tropical East Africa to the south-eastern coast of South Africa. The remaining three species have narrower ranges that overlap with that of *Acokanthera oppositifolia*. The nomenclatural history of these taxa is too lengthy to be summarized here, although two illegitimate names bear explication. The name *Acokanthera ouabaio* (alternatively spelled *Acocanthera wabajo*) is a synonym of *Acokanthera schimperi*; its epithet is both a European adaptation of the Somali word for this taxon (Kupicha 1982), and is the basis for the name given to the highly toxic cardiac glycoside extracted from this and other *Acokanthera* species—ouabain. The name *Acokanthera longiflora* is a synonym of *Acokanthera oppositifolia*; publications enumerating other cytotoxic cardiac glycosides (acovenoside A, acolongifloroside K) from the genus use this name (Kingston and Reichstein 1974; Cassels 1985).

The angiosperm genera *Sclerocarya* (Spondoideae; Anacardiaceae) and *Commiphora* (Bursereae; Burseraceae) derive from closely-related families of resinous, woody trees and shrubs that produce a range of toxic phenolic compounds and terpenoids respectively. These compound mediate plant-herbivore interactions. One compound, alkylcatechol (e.g., urushiol) may cause severe allergenic responses in vertebrates, especially humans, but acute toxicity of these compounds appears to be limited to invertebrates. *Sclerocarya birrea* is commonly cultivated for its edible fruit and bark whose decoction is used for medicinal purposes. As a member of the Spondoideae, it lacks the toxic phenolic compounds (e.g., biflavonoids, alkylcatechols and alkylresorcinols) (Aguilar-Ortigoza and Sosa 2004) that mediate insect interactions as they do in other anacardiaceous genera (e.g., *Calophya* and *Schinus*, Burckhardt and Basset 2000). Nevertheless, reports of the “insecticial” properties of its leaves, bark, and fruits do suggest that *Sclerocarya birrea* has effective chemical defenses, such as high-levels of tannins or flavonoids, that have been documented in its tissues (Prinsloo and Street 2013). *Commiphora* species including *Commiphora africana* and *Commiphora angolensis*, produce gum-oleoresins in stem, leaf, and fruit tissues, that may contain a range of volatile and non-volatile compounds, predominantly terpenoids, with well-documented biological activities (Langenheim 1994). Volatile compounds documented for *Commiphora*, such as the monoterpenes pinene and limonene, the sequiterpene cadiene, and the phenolic compound eugenol, are known to be toxic to insects (Langenheim 1994, 2003). The resins of some species, including that of *Commiphora africana*, are used by indigenous people to repel termites and ticks. Other compounds have an effect on human physiology: sesquiterpenes that interact with the brain’s opiate pathways producing an analgesic effect, and guggulsterones lower the blood lipid content. Phytochemical assays of *Commiphora* species have uncovered a range of unexpected compounds, including phellamurin, which is a dihydroflavonal that mediates butterfly oviposition on Rutaceae (Ma et al. 2005).

*What is ouabain*? Different authors have used ‘ouabain’ to describe the toxic latex from plant sources—*Acokanthera*, *Haemanthus*, *Buphane*, and *Euphorbia* (Arnott 1853; Burton 1856; Hilton et al. 1865; Bolton 1906; Castellani and Chalmers 1919; Fuller 1920; Hall and Whitehead 1927; Reichstein 1965; Cassels 1985). The term is widely used in Africa, from the southern San to the Maasai in Kenya. There is even a plant with the specific name *Acokanthera ouabaio* (Franch. et Poiss.) Cathel. and there are compounds called “ouabain equivalents” (Neuwinger 1974a, b). *Acokanthera* trees and shrubs are a widely-used source of arrow poison and some species are known to have several cardenolides (e.g., Kingston and Reichstein 1974); ouabain may be a cardiac glycoside. In western Zimbabwe, the Matopos Bushmen make arrow poisons from *Acokanthera oppositifolia* (David Cummings, pers. comm. 2014). *Acokanthera* is also used by the Bemba and the Gwembe Tonga in Zambia (Ted Scudder, pers. comm. 2014) and Bushmen in southern Zimbabwe and eastern Botswana (Dornan 1925). According to Ndebele informants in Zimbabwe, the bark of the root of *Acokanthera oppositifolia* is used as a poison and as a means of ensuring that the poison stays on their arrow or spear. *Acokanthera oppositifolia* is more widespread in southern Africa than *Acocanthera oblongifolia*, which is limited to coastal areas. *Acokanthera schimperi* ranges from Bulawayo to Plumtree, western Zimbabwe. Both the Ndebele and Bushmen in western Zimbabwe claimed that their ancestors used *Acokanthera oppositifolia* and *Acokanthera schimperi* for poisoning projectiles (Parry 2007; Hitchcock et al. 2014). Some Bushmen also said that they used snake poison (e.g. from mamba, cobra, and puff adder) combined with *Acokanthera oppositifolia* and *Acokanthera schimperi* as a binding agent. The efficacy of the poison is varied; some informants said that arrow poison took ‘a few minutes to a few hours’ to kill a springbok or impala, from 8–10 hours to kill an eland, and 1–3 days for a giraffe. Parker and Amin (1983) report that the Wata, from the Tsavo area, Kenya, use *Acokanthera*-poisoned arrows to hunt elephants.

*Potential pharmacology of San arrow poisons.* It is fair to ask if highly toxic compounds like diamphotoxin, *Adenium* extracts, and other indigenous poisons have pharmaceutical potential. The San have experience with pharmaceutical bio-prospecting. They are known to chew pieces of the *Hoodia* “cactus” plant (Apocynaceae: *Hoodia gordonii* (Masson) Sweet ex Decne., 1844) to suppress hunger and thirst for long treks. In 1997, the South African Council for Scientific and Industrial Research (CSIR) licensed the UK-based company, Phytopharm, to develop a natural drug. Phytopharm then worked with Pfizer to commercialize and market a drug with the active ingredient, P57. The international outcry led the South African San Council to a court battle with these companies, and eventually to a landmark agreement to share profits with the San Hoodia Benefit Trust (Kahn 2002; www.scienceafrica.co.za/2003/may/san.htm, accessed 25 July 2014). Thus, any exploration of the pharmacology of arrow poisons must be discussed with the San community.

## Conclusions

Under a harsh and drying climate across southern Africa, the San emerged and diversified into numerous distinct communities. Their survival has depended on a profoundly intimate knowledge of their environment—the distribution of all resources (water, tubers, animals) and the availability of the few material resources they keep (e.g., plants for temporary huts, digging sticks, bows and arrows; ostrich eggs for water and pupae; sources of poisons). Under specific local conditions, isolated communities appear to have developed their own specialized poison use and preparation. We have confirmed the species and life stages used as arrow poisons for two San groups, Hai||om and Ju|’hoan, and documented their poison preparation methods. Ethnological data collection such as ours, including the collection of terms in the local vernacular, can open new avenues of research about variations in ecology, fauna and flora. Differences in material culture, due to individual/group style and/or area-specific patterns, *sensu* Wiessner (1983, 1984), may apply to bow and arrow construction and poison preparation. Through this prism we should also expect variations, innovations, and evolution in music, dance, stories, self-decoration, and material culture. However, we propose that the ecological boundaries of the poison cultural practices are severely demarcated, and the poison beetle practice may be strongly conserved, because of the crucial role it plays in food acquisition.

The hunter-gatherer phase of human evolution originated about seven million years ago, and today persists in a few cultures that are fast disappearing under the wheels of modern life. Bow and arrow hunting, a hallmark of hunter-gatherer living, is considered obsolete by some and has become illegal, neglected, or abandoned. Today hunting with traditional weapons is legally permitted only in the Nyae Nyae Conservancy, Namibia. The loss of San cultural knowledge is a proxy for multiple losses—of the environmental context of certain practices (e.g., which plants are nutritious, deadly, or medicinal), of languages, and in transformation of technology (e.g., from blow-darts to guns). Active Hai||om hunters are rare, reflecting their historical eviction from their Etosha homeland in 1954 (Suzman 2004; Dieckmann 2007) and the illegal status of their traditional hunting. Ju|’hoan traditional hunting is maintained today because they have a self-governing conservancy where they can hunt. In these two San communities knowledge is not being passed on to younger generations to sustain future practices.

While the term “San” describes many indigenous groups that share tongue-clicking languages, it is important to keep in mind that there are many sub-cultural differences among these communities. Such subtle differences exhibit the richness of indigenous human societies, provide insight into key innovations in early human behavior, and reflect the ecological context that drives the origins and diversification of traditions and practices. Confusing nomenclature of San communities, their plants and plant compounds, and the beetles and beetle compounds has led to errors in identifications and communication among scientists within and across disciplines. Although these San communities live short distances apart, their arrow poisons are diverse, pointing to an incredibly intimate knowledge of their environment. The discovery of arrow poisons was a significant evolutionary step for humankind, yet we may be facing the last opportunity to document arrow-poison use in southern African hunter-gatherer societies.

## Acknowledgments

We thank the people and governments of Namibia and Botswana for permissions to conduct research. CSC is indebted to David Grimaldi and Robert Goelet (American Museum of Natural History) for supporting the Namibia expedition and to the KU Department of Ecology and Evolutionary Biology General Research Fund grant for supporting manuscript preparation. CSC also thanks colleagues from the National Museum of Namibia—Tharina Bird for logistical arrangements, Holger Vollbrecht and Michael Kazondunge for field assistance, and Eugene Marais for pointing out relevant literature—and John Irish (National Botanical Research Institute, Namibia) for plant identification. We thank Elizabeth Grobbelaar (South African Collection of Insects, ARC-PPRI), for translating Afrikaans text, collaborative fieldwork in South Africa, and photographs. We also thank Fernando Merino for field assistance in Namibia, and Aagje Ashe, Laura Breitkreuz, and Rudolf Jander (University of Kansas) for translating German text. Megan Biesele and Robert K. Hitchcock thank the late Kxao Moses ≠Oma, Tsamkxao ≠Oma, Leon Tsamkxao, |’Angn!ao |Un (Kiewit), |Kunta, N!ae, Dries Alberts, Stacey Main Alberts, Wayne Babchuk, Ben Begbie-Clench, Alison Brooks, Marieka Brouwer Burg, Mary Brown, Alec Campbell, Roger Collinson, Aron Crowell, Ute Dieckmann, Lara Diez, Jim Ebert, Jumanda Gakelebone, John Hardbattle, Stasja Koot, Kadison Khomob, Melinda Kelly, Steve Lawry, Richard Lee, Willemien LeRoux, Judith Miller, Michael Murphy, Ashton Murwira, Alan Osborn, Michael Painter, Larry Robbins, Beatrice Sandelowsky, Maria Sapignoli, Roy Sesana, George Silberbauer, Axel Thoma, Helga Vierich, Diana Vinding, Nick Walker, Thomas Widlok, Polly Wiessner, the U.S.National Science Foundation, the Ministry of Environment and Tourism (Namibia), Millennium Challenge Corporation, the U.S. Agency for International Development, the Department of Wildlife and National Parks (Botswana), the Nyae Nyae Conservancy, the Nyae Nyae Development Foundation of Namibia, and the Cgae Cgae Tlhabololo Development Trust (Botswana). We thank David Furth, Alexander Konstantinov, and Claire Smith (USNM) for supplying photos of *Blepharida* and Linda Trueb, Leonard Krishtalka, Matthias Schöller for commenting on an earlier draft of this manuscript. Finally we thank reviewers Elizabeth Grobbelaar, Bill Shepard, Clarke Scholtz, and Alexander Konstantinov and anonymous reviewers for suggestions that improved the final manuscript.
